# Targeting non-coding RNAs to overcome osimertinib resistance in *EGFR*-mutated non-small cell lung cancer

**DOI:** 10.3389/fonc.2024.1442237

**Published:** 2024-09-11

**Authors:** Beilei Zeng, Kelun Gan, Yuanhang Yu, Jianping Hu, Qiao Deng, Chong Yin, Xi Gao

**Affiliations:** ^1^ Department of Oncology, Affiliated Hospital of North Sichuan Medical College, Nanchong, Sichuan, China; ^2^ School of Medical Imaging, North Sichuan Medical College, Nanchong, Sichuan, China; ^3^ Department of Clinical Laboratory, Affiliated Hospital of North Sichuan Medical College, Nanchong, Sichuan, China; ^4^ Department of Laboratory Medicine, Translational Medicine Research Center, North Sichuan Medical College, Nanchong, Sichuan, China

**Keywords:** non-small cell lung cancer, EGFR, ncRNA, osimertinib resistance, mechanism

## Abstract

Osimertinib, a third-generation inhibitor of epidermal growth factor receptor (EGFR) tyrosine kinase, exhibits remarkable efficacy in prolonging the survival of patients with non-small cell lung cancer (NSCLC) carrying *EGFR* mutations, surpassing the efficacy of first- and second-generation EGFR tyrosine kinases. Nevertheless, the emergence of osimertinib resistance is inevitable, necessitating an investigation into the underlying mechanisms. Increasing evidence has revealed that non-coding RNAs (ncRNAs), including microRNAs, long ncRNAs, and circular RNAs, play a significant role in the development and progression of lung cancer. These ncRNAs regulate essential signaling pathways, offering a novel avenue for understanding the fundamental mechanisms of osimertinib resistance. Recent studies have reported the significant impact of ncRNAs on osimertinib resistance, achieved through various mechanisms that modulate treatment sensitivity. We provide a concise overview of the functions and underlying mechanisms of extensively researched ncRNAs in the development of osimertinib resistance and emphasize their potential clinical application in *EGFR*-mutated NSCLC resistant to osimertinib. Finally, we discuss the obstacles that must be addressed to effectively translate ncRNA-based approaches into clinical practice.

## 1 Introduction

Non-small cell lung cancer (NSCLC) is responsible for approximately 85% of all occurrences of lung cancer, which is the primary cause of cancer-related fatalities globally, as reported in recent studies (1, 2). The two primary histological phenotypes of NSCLC are adenocarcinoma and squamous cell carcinoma (3). Traditionally, the primary approach for NSCLC management involves combination chemotherapy protocols that incorporate platinum-based medications. However, despite these treatments, the mortality rate among patients with NSCLC remains high.

Genome sequencing is essential for finding mutant genes linked to NSCLC development, therapeutic resistance, and prognosis. Molecular alterations in NSCLC encompass mutations in the epidermal growth factor receptor (*EGFR*) and *BRAF* (specifically the V600E mutation) genes and the Kirsten rat sarcoma viral oncogene (*KRAS*); rearrangements in the anaplastic lymphoma kinase (*ALK*) gene and ROS proto-oncogene receptor tyrosine kinase 1 (*ROS1*); alterations in exon 14 of the mesenchymal-epithelial transition (*MET*) gene; and gene fusions involving neurotrophic receptor tyrosine kinase, among others (2, 4, 5). Among the mutant genes, *EGFR* undergoes a significant number of mutations, with a prevalence of up to 59.4% in Asian female nonsmoking patients with NSCLC. *EGFR* exon 19 deletions and exon 21 L858R point mutations, known as classical activating mutations, make up 90% of all mutations (2). Targeted therapy that specifically focuses on *EGFR* has shown significant benefits compared to conventional chemotherapy and radiotherapy treatments in patients with lung malignancies harboring *EGFR*-activating mutations (2), leading to profound changes in treatment approaches and research strategies for lung cancer (2). Despite the high initial effectiveness of first-line EGFR tyrosine kinase (TKI), the majority of individuals undergo cancer progression within a timeframe of 9 to 13 months after receiving therapy (6). The *EGFR* T790M mutation is the predominant cause of resistance to first- or second-generation EGFR-TKIs, affecting approximately 50%–60% of patients (4, 6).

Osimertinib (AZD9291) was designed with the specific purpose of selectively targeting the *EGFR* T790M mutation. Clinical evidence from the AURA serial studies confirms osimertinib as an efficacious treatment option with a satisfactory safety profile for patients with advanced NSCLC who possess classical T790M and *EGFR*-activating mutations. Owing to the promising outcomes shown in the AURA serial studies, osimertinib was the first third-generation EGFR-TKI to be clinically approved for treating patients with NSCLC harboring the *EGFR* T790M mutation (4, 6). In addition, findings from the FLAURA study led to osimertinib’s approval as the initial therapy for patients with advanced NSCLC with *EGFR*-activating mutations, irrespective of their T790M status (7, 8). Nevertheless, osimertinib resistance is a well-established clinical phenomenon, irrespective of whether it is administered as a first-line or second-line treatment, which greatly hampers its long-term therapeutic advantages.

The mechanisms underlying osimertinib resistance, which may be associated with tumor evolution, clonal heterogeneity, and selection pressure, can be categorized into two types: EGFR-dependent (on-target) and EGFR-independent (off-target) mechanisms (9). On-target mechanisms that are specifically targeted entail changes in important amino acid residues that physically obstruct osimertinib binding to the ATP-binding site of the EGFR-TKI domain, ultimately leading to treatment resistance, such as the absence of the T790M mutation and the acquisition of *EGFR* mutations (e.g., C797X, G796X, L927H, EGFR amp). EGFR-independent pathways are more prevalent and consequential in osimertinib resistance compared to EGFR-dependent mechanisms, possibly because of the superior on-target inhibition of osimertinib. These mechanisms include modifications in other TKI receptors (e.g., AXL), activation of bypass pathways (e.g., *c-Met* amplification, *HER2* amplification, *FGFR1* amplification), acquired oncogenic fusions (e.g., *RET*, *ALK*, *BRAF*), activation of downstream signaling pathways (e.g., PI3K/AKT, JAK/STAT, RAS/MAPK/ERK), histologic and phenotypic transformations (e.g., epithelial-mesenchymal transition [EMT] and squamous cell carcinoma and SCLC transformation) (9–11). Despite the identification of certain resistance mechanisms to osimertinib, comprehension of 30%–50% of these mechanisms remains lacking (9). Moreover, resistance mechanisms vary depending on whether osimertinib is administered initially or in patients whose tumors have developed the *EGFR* T790M resistance mutation while being treated with first- or second-generation EGFR-TKIs (9). Therefore, investigating a novel resistance mechanism and identifying potential therapeutic interventions is imperative to overcome the obstacles posed by osimertinib resistance.

## 2 Overview of non-coding RNAs

NcRNAs, constituting over 90% of the RNAs produced by the human genome, cannot produce proteins or peptides (12, 13). The ncRNAs research is continuously evolving, with categorization based on their length, form, and location. These classes encompass intronic RNAs, microRNAs (miRNAs), long ncRNAs (lncRNAs), circular RNAs (circRNAs), and extracellular RNAs (12). Among these, miRNAs, lncRNAs, and circRNAs, termed classic ncRNAs, have been extensively studied due to their significant functions in controlling the onset and progression of different types of malignancies (14, 15).

miRNAs are a group of short ncRNAs, usually 20–22 nucleotides long, arising from larger stem-loop structures, and can suppress the activity of mRNA by binding to them (16). Their generation involves a series of complex processes, including transcription into primary miRNAs and nuclear processing by Drosha and Dgcr8, resulting in the formation of precursor miRNAs. After precursor miRNAs are delivered to the cytoplasm, they are cleaved to generate miRNA-miRNA duplexes, where one of the two miRNAs exerts an inhibitory function and the other undergoes degradation (17). A single miRNA can target various mRNAs, while several different miRNAs can target a single mRNA. Moreover, miRNAs play vital roles in many essential biological processes, and their unique activities in distinct tissues have been demonstrated (15). Recently, miRNAs have garnered significant attention in cancer research for their dysregulation’s impact on the ability of cancer cells to resist traditional chemotherapy and new targeted therapies (18).

LncRNAs are the most intricate ncRNA subtype and are characterized by RNA molecules over 200 nucleotides in length that often do not undergo protein translation (14). Predominantly distributed within the nucleus, they are transcribed by RNA polymerase II from independent promoters, similar to mRNA production, and have a length comparable to that of mRNA, albeit with fewer exons. The process of lncRNA biogenesis closely resembles that of mRNA biogenesis, with certain modifications to the underlying mechanisms (19). LncRNAs exhibit a high tissue-specific expression pattern and carry out cis-regulatory actions at their transcription sites as well as trans-acting roles at a distance from the transcription sites (13, 14). In contrast to small ncRNAs, lncRNAs have a wide range of functional mechanisms; they can affect gene expression at many stages, including epigenetic, transcriptional, and post-transcriptional processes (13). In addition to directly binding with DNA, lncRNAs can also interact with a diverse range of other RNA molecules, such as mRNAs, circRNAs, and miRNAs (15). Multiple studies have demonstrated that lncRNAs have significant regulatory functions in tumor development, metastasis, and drug resistance (13, 14, 20).

CircRNAs are a unique type of ncRNA that possess a unique structure, forming closed loops with no interruptions where the 3’ and 5’ ends are connected. The circRNA’s covalently closed loop structure is formed via a process called “back-splicing,” in which a downstream splice donor is coupled to an upstream splice acceptor. This enhances their stability and resistance to exonuclease degradation compared to linear RNAs (21). CircRNAs function as molecular decoys for miRNAs, disrupting gene transcription and translation processes. They may also prolong the stability of certain mRNA molecules (14) and exhibit an intricate and variable expression profile (15, 22) by participating in development, cell cycle regulation, stress responses, and apoptosis, among other processes. Accumulating evidence underscores circRNAs’ substantial impact on human malignancies and can function as dependable biomarkers (15, 23).

## 3 Link between ncRNAs and *EGFR*-mutated NSCLC

Multiple investigations conducted over the course of several years have established a clear association between ncRNAs and lung cancer (24, 25). For instance, members of the miR-34 family negatively regulate the development of lung squamous carcinoma and lung adenocarcinoma (LUAD) (26, 27). In addition, Zhao et al. demonstrated a direct relationship between the concentrations of miR-34a and miR-34c in both plasma and tumor tissue. They also found that a decreased level of circulating miR-34a and miR-34c is associated with a negative prognosis in patients with NSCLC (28). Metastasis-associated lung adenocarcinoma transcript 1 is a well-studied lncRNA in the field of cancer, serving as a diagnostic, prognostic, and therapeutic indicator in lung cancer (29). These findings provide a theoretical basis for NSCLC treatment and the identification of potential biomarkers for predicting its prognosis.

In *EGFR*-mutated NSCLC, ncRNAs hold promise as biomarkers for identifying EGFR status and monitoring EGFR-TKI treatment effectiveness. Weiss et al. demonstrated that miR-128b can inhibit EGFR protein expression (30). Based on 105 female non-smokers with LUAD, Zhang et al. confirmed that circulating miR-122 and miR-195 in plasma may predict the *EGFR* mutation status (31). Similarly, Qu et al. identified circulating miR-122 as a indicator for the *EGFR* p.L858R mutation and miR-195 for EGFR exon 19 deletion, with circulating miR-107 potentially serving as a marker for both EGFR exon 19 deletions and *EGFR* p.L858R mutations (32). Utilizing the Clariom D Human Chip technology, the expression patterns of lncRNAs in pleural effusions were examined in three patients with *EGFR* mutations and three patients without *EGFR* mutations. The analysis revealed five lncRNAs (FTH1P2, metastasis-associated lung adenocarcinoma transcript 1, NONHSAT051892, NONHSAT017369, and SCARNA7) were significantly correlated with *EGFR* mutations (33). In an osimertinib-resistant cell line model, Zhang et al. discovered that several miRNAs, including miR-19b-1-5p, miR-34a-5p, miR-219a-5p, miR-708-3p, miR-708-5p, miR-7704, and miR-10395-3p, are potentially significant in osimertinib resistance development (34). Vadla et al. revealed that the modulation of the NSCLC response to osimertinib is presumably influenced by extracellular vesicles (EV) (miR-184, let-7b-5p) and circulating (miR-22-3p) plasma miRNAs in H1975 NSCLC cells (35). Additionally, Chen et al. reported that circANKRD11, circMUC16, circRYK, circNAV3, circMKLN1, circMUC16, circPPP4R1, circSPAG16, and circMYH9 were upregulated in osimertinib–resistant cell lines, whereas circRSF1, circPDLIM5, circGLS, circMIB1, circOMA1, circSNX13, circPLOD2, circTNFRSF21, circDCBLD2, circMPP6, and circSATB2 were downregulated (36).

## 4 Mechanisms of osimertinib resistance mediated by ncRNAs

The primary mechanisms through which acquired resistance to osimertinib is mediated by ncRNAs include aberrant activation of bypass and downstream signaling pathways, abnormal modulation of EMT, aberrant modulation of metabolic pathways, interference with apoptosis, and pyroptosis. The main mechanisms are discussed below and summarized in **Table 1**.

**Table 1:** List of ncRNA-related mechanisms of osimertinib resistance in *EGFR*-mutated NSCLC.

Human ncRNAs	Function	Molecular mechanisms and targets	References
circ_0005576	oncogene	Interacts with miR-512-5p and increases insulin-like growth factor receptor (IGF1R) levels	(22)
circ_PPAPDC1A	oncogene	Targets miR-30a-3p and increases the IGF1R levels	(37)
lncRNA TSLNC8	suppressor gene	Inhibits the EGFR-STAT3 pathway and significantly enhances the therapeutic effects of osimertinib	(38)
lncRNA HIF1A-AS2	oncogene	Activates the IL-6/STAT3 axis by inhibiting miR-146b-5p expression	(39)
miR-146b-5p	suppressor gene	Suppresses interleukin-1 receptor-associated kinase 1 (IRAK1) expression, resulting in NF-κB activity inhibition and the production of NF-κB-associated IL-6 and IL-8	(40)
miR-205-5p, miR-203a-3p, miR-200c-3p, and miR-183-5p	suppressor gene	Associated with epithelial-mesenchymal transition (EMT); target prediction analysis identified ZEB1 as a crucial target for these miRNAs	(41)
let-7c	suppressor gene	Reverses EMT and increases the sensitivity of cells to osimertinib by upregulating E-cadherin and downregulating ZEB1	(42)
MiR-200c	suppressor gene	Controls EMT by reducing ZEB1 expression and increasing E-cadherin expression	(43)
lncRNA CASC8	suppressor gene	Enhances FOXM1 activity and enhances EMT	(44)
miR-210-3p	oncogene	Prompts EMT by inhibiting E-cadherin expression and enhancing vimentin expression	(45)
miR-147b	oncogene	Disrupts the tricarboxylic acid (TCA) cycle and triggers pseudohypoxia reactions	(46)
miR-21-3p and miR-21-5p	oncogene	Dysregulates purine metabolism and oxidative stress pathways by suppressing adenylosuccinate lyase (ADSL)	(47)
hsa_circ_0002130	oncogene	Inhibits miR-498 and increases the key glycolysis factors, including lactate dehydrogenase A (LDHA), hexokinase 2 (HK2), and glucose transporter 1 (GLUT1)	(48)
circ7312	oncogene	Decreases pyroptosis and apoptosis through interaction with the miR-764/MAPK1 axis	(49)
lncRNA CRNDE	oncogene	Decreases the eIF4A/MUC1/EGFR pathway and impairs apoptotic activity	(50)
lnc-MZT2A-5:1	oncogene	Stimulates the activation of MRC-5 fibroblast cells	(51)
circKRT17	oncogene	Modified by METTL3, enhancing the nuclear transport and stability of YAP1 by recruiting EIF4A3	(52)
let-7b	suppressor gene	METTL3 decreases pri-Let-7b and increases both the expression of pre-let-7b and mature let-7b, thereby suppressing Notch signaling pathway activation	(53)
circFBXW7	suppressor gene	Represses Wnt pathway functions through the activity of its short polypeptides, circFBXW7-185AA	(54)
circRBM33	oncogene	Modulates the DNMT1/IL-6 axis	(55)
lnc-TMEM132D-AS1	oncogene	Binds to miR-766-5p, resulting in the increased expression of ectonucleoside triphosphate diphosphohydrolase-1 (ENTPD1)	(56)

### 4.1 Aberrant activation of bypass and downstream signaling pathways

Type 1 insulin-like growth factor receptor (IGF1R), a member of the insulin receptor family, is a class II receptor TKI that plays a significant function in cell growth and differentiation (57). A previous study has suggested a correlation between IGF1R deregulation and the differentiation and survival of individuals with LUAD (58). In *EGFR*-mutant NSCLC cells carrying the T790M mutation, IGF1R activation can lead to the development of osimertinib resistance (59). Liu et al. demonstrated that hsa_circ_0005576 acts as a miRNA sponge by directly interacting with miR-512-5p (22), and Tang et al. proved that circ_PPAPDC1A acts as a miRNA sponge targeting miR-30a-3p (37), both increasing the IGF1R levels, thus contributing to LUAD cells’ resistance to osimertinib. Targeting hsa_circ_0005576 and circ_PPAPDC1A can inhibit IGFR1 expression and reverse osimertinib resistance (22, 37).

In addition, the signal transducer and activator of transcription 3 (STAT3), a critical EGFR downstream signaling pathway molecule, significantly regulates various cellular processes, such as proliferation, differentiation, apoptosis, and immune response. It is also associated with EGFR-TKI resistance in lung cancer (60). Therefore, targeting STAT3 could reverse acquired resistance to EGFR-TKI. Overexpression of TSLNC8 can inhibit the EGFR-STAT3 pathway and greatly enhance the therapeutic effects of osimertinib (38). Similarly, the lncRNA HIF1A-AS2 can enhance resistance to osimertinib by modulating the miR-146b-5p/IL-6/STAT3 axis in LUAD (39).

Activation of NF-κB is a crucial mechanism that contributes to tumorigenesis in multiple solid tumors, including lung cancer (61). Recent research indicates EGFR-TKIs trigger NF-κB activation, leading to the emergence of EGFR-TKIs resistance via several pathways such as activation-induced cytidine deaminase, Akt, and interleukin-6 (IL-6)/JAK2/STAT3 in NSCLC (62). MiR-146b-5p suppresses interleukin-1 receptor-associated kinase 1 expression, thereby inhibiting NF-κB activity and subsequent production of NF-κB-associated IL-6 and IL-8 in two primary EGFR T790M-preserved osimertinib-resistant cancer cell lines, PE2988 and PE3479, and enhanced EGFR TKI-induced apoptosis (40). This enhanced apoptosis suggests the potential of miR-146b-5p as an efficient approach for addressing EGFR-TKI resistance.

### 4.2 Aberrant modulation of EMT

During EMT, epithelial cells lose their apical-basal polarity and acquire characteristics that are typically associated with mesenchymal cells (41). It is widely recognized that the mesenchymal phenotype drives tumor progression and is associated with resistance against anticancer therapy (63). Previous research has shown that NSCLC cells developing resistance to gefitinib or osimertinib display EMT characteristics without secondary *EGFR* mutations. Inhibiting EMT has shown promise in enhancing sensitivity to EGFR-TKIs in EGFR resistance cell lines (64). Preliminary studies suggest that ncRNAs act as key modulator of EMT by regulating EMT transcription factors, including the TWIST, SNAIL, and ZEB families (41, 65). The miR-205-5p, miR-203a-3p, miR-200c-3p, and miR-183-5p, which originate from extracellular vesicles, are associated with EMT. Furthermore, their anticipated target ZEB1 is notably upregulated in EGFR-TKI resistant cells, including osimertinib-resistant cells (41).

Regarding ncRNAs’ function in controlling EMT and its impact on osimertinib resistance, Li et al. provided evidence that the let-7c molecule has a notable impact on the process of EMT and is linked to osimertinib resistance in NSCLC cells carrying *EGFR* T790M mutation. Elevated let-7c expression effectively reversed EMT, enhancing cells’ sensitivity to osimertinib by upregulating E-cadherin and downregulating ZEB1 expression, and vice versa. Additionally, let-7c can also impair the cancer stem cell phenotype by suppressing WNT1 and TCF-4 (42). MiR-200c is also critical in controlling EMT by reducing ZEB1 expression (66). Fukuda et al. observed significantly reduced miR-200c expression in osimertinib-resistant lung cancer cells compared to their parental cells. The overexpression of miR-200c resulted in a decrease in ZEB1 expression and an increase in E-cadherin expression in the resistant cells (43). Recently, it was reported that lncRNA cancer susceptibility candidate 8 is highly expressed in NSCLC and has a critical function in enhancing the growth, migration, and invasion of NSCLC cells, contributing to osimertinib resistance. Cancer susceptibility candidate 8 modulates the function of genes that participate in the EMT process by enhancing FOXM1 activity. Suppression of cancer susceptibility candidate 8 expression has been linked to increased osimertinib sensitivity (44). In addition, there is an increasing comprehension of the function of exosomal ncRNAs in promoting EMT and the development of treatment resistance in cancer (67). Exosomes can be released from drug-resistant cells, transferring factors causing resistance to drug-sensitive cells, resulting in the emergence of a drug-resistant characteristic (67). Co-cultivation of osimertinib-sensitive cells with osimertinib-resistant cells leads to the induction of *EMT* alterations in osimertinib-sensitive cells. For example, miR-210-3p could potentially be an important substance carried by exosomes originating from osimertinib-resistant NSCLC cells, impacting EMT and drug resistance in osimertinib-sensitive cells (45).

### 4.3 Aberrant modulation of metabolic pathways

Aberrantly regulated cancer metabolism, characterized by aerobic glycolysis and heightened anabolic pathways, plays crucial roles in tumorigenesis, metastasis, and drug resistance (68). The TCA cycle is an essential metabolic process. Activation of pseudohypoxic responses, which induce the production of hypoxia-associated proteins regardless of oxygen levels, can dysregulate the TCA cycle, contributing to cancer development and treatment resistance (46). Elevated miR-147b levels facilitate this process by disrupting the TCA cycle and triggering pseudo-hypoxic reactions. Inhibition of miR-147b and the associated downstream TCA cycle and/or hypoxic pathways may offer a novel approach for mitigating osimertinib resistance in NSCLC (46).

Additionally, cancer cells use a tolerance process to shield themselves from the effects of EGFR inhibitors. Drug-tolerant persister cells play a crucial role and are a probable cause of treatment resistance. Therefore, eradicating persister cells is imperative to enhance the response to antitumor therapy (69). Zhang et al. discovered the mechanism by which tumor cells transition into the DTP state through the interplay of miRNAs and metabolic pathways. According to the study, miR-21-3p and miR-21-5p levels were elevated in *EGFR*-mutant lung cancer cells tolerant to EGFR-TKI treatment. This increase is achieved by dysregulating purine metabolism and oxidative stress pathways through the suppression of adenylosuccinate lyase, an important enzyme in purine metabolism. Targeting miR-21 partially restores alterations in purine metabolites and oxidative stress in drug-tolerant persister cells, thus mitigating the development of drug resistance to anti-EGFR treatment (47).

Aerobic glycolysis, referred to as the Warburg effect, is a metabolic process that promotes cell growth, metastasis, and treatment resistance in tumor cells. This process involves increased synthesis of lactate and consumption of glucose, representing a hallmark of cancer cells in humans (48, 70). Recent studies have revealed a substantial increase in glycolysis in osimertinib-resistant NSCLC cells. Furthermore, proteins associated with glycolysis, such as lactate dehydrogenase A, hexokinase 2, and glucose transporter 1, exhibit notably increased expression in osimertinib-resistant NSCLC cells. Targeting glycolysis may present a new and effective approach to addressing the challenge of osimertinib resistance. Ma et al. showed that hsa_circ_0002130 exhibits significant upregulation in osimertinib-resistant NSCLC cells. Furthermore, hsa_circ_0002130 could increase the expression of lactate dehydrogenase A, hexokinase 2, and glucose transporter 1 in osimertinib-resistant NSCLC cells by specifically inhibiting miR-498. Therefore, targeting hsa_circ_0002130 may be a novel mechanism to overcome osimertinib resistance (48).

### 4.4 Interference with apoptosis and pyroptosis

In multicellular organisms, the maintenance of ontogeny, homeostasis, and pathological processes relies on the intricate interplay between cell proliferation, differentiation, and death. There are two primary classifications of cell death: necrosis and programmed cell death. Apoptosis is a form of programmed cell death characterized by the spontaneous self-destruction of cells and is regulated by specific genes. During this process, the cell membrane remains intact and does not trigger inflammation. Pyroptosis is now widely recognized as a type of cellular death. Similar to apoptotic cells, pyroptotic cells experience nuclear condensation and chromatin DNA destruction, maintaining positive terminal deoxynucleotidyl transferase dUTP nick end labeling staining (71). The significance of apoptosis and pyroptosis in tumorigenesis and therapeutic response has gained considerable attention in recent literature (71, 72).

Multiple studies have demonstrated that ncRNAs can impact the efficacy of osimertinib treatment in NSCLC with *EGFR* mutations by regulating cancer cell apoptosis and pyroptosis, either individually or in combination. For instance, Dai et al. conducted a study that showed how circ7312 can enhance osimertinib resistance in PC9/ER cells. This is achieved by decreasing pyroptosis and apoptosis by interacting with the miR-764/MAPK1 axis, thereby enhancing osimertinib resistance (49). Moreover, the lncRNA colorectal neoplasia differentially expressed has been implicated in impairing apoptotic activity and promoting resistance to osimertinib and afatinib via the eIF4A/MUC1/EGFR pathway in (osimertinib and afatinib-resistant) *EGFR*-mutated lung cancer cells (50).

### 4.5 Other mechanisms

Exosomes are extracellular vesicles with sizes ranging from 40 nm to 160 nm and released by numerous cells in the tumor microenvironment (73). Tumor-derived exosomes have a vital function in facilitating communication between cancer cells and their surrounding environment, impacting the development and progression of several cancer processes, including drug resistance (74). Exosomal lnc-MZT2A-5:1, derived from AZD9291-resistant NSCLC cell lines, has the ability to enhance the activation of MRC-5 fibroblast cells, which are a significant cell type in the tumor microenvironment and have a vital role in the malignant characteristics of NSCLC. Therefore, it may offer a possible target for addressing resistance to AZD9291 and presenting a therapeutic opportunity (51).

N6-methyladenosine (m6A) is a prevalent RNA modification observed in eukaryotes, primarily in messenger RNA. Growing evidence suggests that m6A modifications can influence the splicing and maturation of ncRNAs and are implicated in the development of cancer (75). RNA methyltransferase methyltransferase-like 3 (METTL3) represents a significant constituent of m6A “writers” (75). Ji et al. reported that METTL3 and circular RNA KRT17 are highly expressed in osimertinib-resistant LUAD tissues and cells. The m6A modification of circular RNA KRT17, facilitated by METTL3, enhanced the nuclear transport and stability of YAP1 by recruiting EIF4A3. This process promotes osimertinib resistance in LUAD (52). Furthermore, the ability of metformin to enhance the sensitivity of osimertinib-resistant cells relies on increased mature let-7b production. This role is also facilitated by METTL3, which reduces the level of promoter methylation of let-7b in osimertinib-resistant HCC827 and H1975 cells, increasing the expression of mature let-7b, which plays a critical role in suppressing Notch signaling (53). In addition, using bioinformatics analysis and array experiments, Li et al. identified significantly low circFBXW7 expression in osimertinib-resistant cell lines. The study reported that circF-BXW7 effectively hinders the capabilities of LUAD stem cells. Additionally, it counteracts resistance to TKIs by regulating the functions of the Wnt pathway. This regulation occurs through the activity of its short polypeptides, circFBXW7-185AA, which increase the ubiquitination of β-catenin and inhibit the activation of the Wnt pathway. This process is regulated by the m6A regulator, YTHDF3 (54). DNMT1 encodes an enzyme that facilitates methyl group transfer to genomic DNA cytosine nucleotides. Several investigations have shown a robust link between abnormal expression and variations in DNMT1 and the occurrence of tumors, metastasis, and treatment resistance. Pan et al. reported significantly higher circRBM33 expression in H1975/OR cells compared to its parental-sensitive cells. Hence, bioinformatics analysis has proven that circRBM33 potentially plays a role in conferring resistance to osimertinib in H1975-OR cells by modulating the DNMT1/IL-6 axis (55).

Wang et al. observed significantly increased expression of lnc-TMEM132D-AS1 in osimertinib-resistant H1975/OR and HCC827/OR cells. The upregulation of lnc-TMEM132D-AS1 may be attributed to its direct binding to miR-766-5p, leading to the upregulation of ectonucleoside triphosphate diphosphohydrolase-1 (56).

## 5 Clinical implications of ncRNA analysis in osimertinib-resistant NSCLC

NcRNAs hold significant clinical relevance as biomarkers in specific cells, tissues, and diseases owing to their specific expression patterns in particular cells, tissues, and developmental stages (14). Numerous studies have assessed ncRNA expression levels in tumor tissues and plasma samples from osimertinib-resistant patients with LUAD to predict the treatment response to osimertinib. The findings of these studies are summarized in **Table 2**. Ji et al. discovered that circular RNA KRT17 levels were higher in osimertinib-insensitive LUAD tissues (n = 16) than in osimertinib-sensitive tissues (n = 24) (52). In addition, substantial quantities of ncRNAs are released as EV and circulating ncRNAs, enabling their non-invasive detection in cancer plasma or serum (76). These characteristics position them as prospective biomarkers for therapy response assessment and drug resistance prediction (77–79).

**Table 2:** Clinical significance of ncRNAs in osimertinib-resistant non-small cell lung cancer.

ncRNAs	Study population	Samples	Clinical findings	References
circKRT17	24 osimertinib-sensitive patients with lung adenocarcinoma (LUAD) and 16 osimertinib-resistant patients with LUAD	Lung cancer tissue	circKRT17 was highly expressed in osimertinib-resistant LUAD tissues	(52)
miR-6513-5p, miR-5189-5p, miR-1468-3p, and miR-323-3p	27 NSCLC patients who developed EGFR T790M following treatment with first or second generation of EGFR-TKI	Plasma samples (the initial osimertinib treatment and PD)	The four miRNAs have the potential to serve as markers for distinguishing between osimertinib-resistant and osimertinib-sensitive patients with NSCLC with a high level of accuracy. They are associated with prolonged overall survival (OS) and time-to-treatment failure (TTF).	(80)
miR-3913-5p and miR-184	67 EGFR-mutated patients with NSCLC	Plasma samples (the initial osimertinib treatment and PD)	There was a marked rise in the levels of exosome-derived miR-3913-5p and miR-184 after the initiation of osimertinib resistance. miR-3913-5p and miR-184 demonstrated greater utility in predicting resistance to osimertinib in patients with EGFR exon 21 L858R point mutations than exon 19 deletion. miR-3913-5p correlated with other tumor parameters, including platelet count, Tumor, Node, Metastasis (TNM) stage, carcinoembryonic antigen tumor marker, and the presence of distant metastases.	(81)
miR-494-3p	21 NSCLC patients with acquired EGFR T790M-mutation following treatment with first or second generation of EGFR-TKI	Plasma samples (the initial osimertinib treatment and PD)	miR-494-3p showed a significant elevation in the plasma sample at the disease progression stage	(82)
lncRNA MSTRG.292666.16	2 NSCLC patients with acquired EGFR T790M-mutation following treatment with first generation of EGFR-TKI	Plasma samples (the initial osimertinib treatment and PD)	MSTRG.292666.16 exhibited a considerable increase in the plasma of patients who were resistant to osimertinib.	(83)
lnc-TMEM132D-AS1	25 osimertinib-sensitive patients with LUAD and 25 osimertinib-resistant patients with LUAD	Plasma samples from patients with NSCLC	lnc-TMEM132D-AS1 in the plasma of patients with NSCLC who developed resistance to osimertinib was approximately 3.52-fold higher than in patients who were sensitive to osimertinib.	(56)
hsa_circ_0002130	32 osimertinib-sensitive patients with LUAD and 28 osimertinib-resistant patients with LUAD	Plasma samples of NSCLC patients	hsa_circ_0002130 exhibited higher expression levels in serum exosomes obtained from patients with osimertinib-resistant NSCLC in comparison to those with osimertinib-sensitive NSCLC	(48)
lncRNA LINC00460	54 patients diagnosed with recurrent post-operative EGFR-mutant LUAD were treated with osimertinib	Lung cancer tissue and plasma samples	The upregulation of LINC00460 resulted in a markedly reduced overall response rate to osimertinib; high LINC00460 expression in the primary tumor site and plasma of patients with EGFR-mutant NSCLC following osimertinib therapy is indicative of reduced PFS and OS	(84)

Janpipatkul et al. demonstrated that four plasma exosomal miRNAs (miR-6513-5p, miR-5189-5p, miR-1468-3p, and miR-323-3p) have the potential to serve as markers to distinguish between patients with osimertinib-resistant and osimertinib-sensitive NSCLC with high accuracy. This was determined by ROC analysis, with area under the curve values of 0.934, 0.928, 0.886, and 0.878, respectively. They also found that patients with overexpression of these four exosomal miRNAs exhibited prolonged overall survival and time-to-treatment failure compared to those without such upregulation, although this reported difference was not statistically significant (80). Li et al. observed a notable increase in the levels of exosome-derived miR-3913-5p and miR-184 expression following the onset of osimertinib resistance in both cell lines and the peripheral blood of patients with NSCLC. The two miRNAs were particularly useful in predicting osimertinib resistance in individuals with *EGFR* exon 21 L858R point mutations when compared to the population with exon 19 deletions (81).

Moreover, a correlation was observed between exosomal miR-3913-5p and other tumor parameters, including platelet count, TNM stage, carcinoembryonic antigen tumor marker, and presence of distant metastases (81). MiR-494-3p, derived from exosomes, was notably increased in plasma samples obtained from a group of 21 patients with NSCLC who had the *EGFR* T790M mutation during the stage of disease progression (82). Regarding lncRNAs and circRNAs, the expression level of exosome-derived MSTRG.292666.16 was markedly increased in the plasma of patients who were resistant to osimertinib (83). Other studies have shown a 3.52-fold higher expression of lnc-TMEM132D-AS1 in the plasma of patients with NSCLC who developed resistance to osimertinib, which was approximately compared to patients who were osimertinib-sensitive (56).

In addition, Ma et al. had previously reported higher expression levels of hsa_circ_0002130 in serum exosomes from patients with osimertinib-resistant NSCLC than in those with osimertinib-sensitive NSCLC (area under the curve=0.792; P<0.001) (48). Nakano et al. showed that upregulation of LINC00460 resulted in a significantly reduced overall response rate to osimertinib (16.6% vs. 60.0%; P=0.044) and high LINC00460 expression in the primary tumor site and plasma of patients with *EGFR*-mutant NSCLC following osimertinib therapy was indicative of reduced progression-free survival and OS (84).

These findings underscore the significance of ncRNA as a robust and non-invasive predictive marker for forecasting osimertinib therapy effectiveness and prognostic value in patients with *EGFR*-mutated NSCLC, thereby aiding therapeutic decision-making processes. Furthermore, it is imperative to conduct extensive clinical trials to assess those robust and non-invasive ncRNAs to provide an objective basis for clinical treatment.

## 6 Conclusion and future challenges

This review comprehensively analyzes the status of ncRNA-mediated osimertinib resistance in *EGFR*-mutant NSCLC. Preclinical studies have extensively explored the application of the exogenous expression of tumor-suppressive ncRNAs or knockdown of oncogenic ncRNAs to reverse osimertinib resistance.

Although these studies offer valuable insights into overcoming osimertinib resistance, the effective implementation of ncRNA-based treatment interventions to tackle osimertinib resistance in clinical settings remains a considerable challenge. Indeed, ncRNA-based therapies have yet to be applied clinically (13, 15). Although several miRNA-based treatments made it to phase II clinical trials for advanced tumors, all of them were ultimately terminated (85). One of the primary factors is the intrinsic instability of ncRNAs, which are prone to rapid degradation by ribonucleases and their subsequent excretion from the body via renal and hepatic mechanisms (85). Although circRNAs possess a covalently closed structure characterized by excellent stability, existing profiling methods are complex and expensive, hampering clinical implementation (23). In addition, following activation of the immune response, the host system perceives exogenous ncRNAs as pathogens. Hence, an autoimmune response of the human body’s immune system may be activated to eradicate these RNAs and impede their site-specific accumulation (85), and immune-related side effects also pose a substantial challenge. Furthermore, it is crucial to carefully evaluate the safety profile of ncRNA-targeted therapeutics due to the possible occurrence of off-target effects that may arise from the control of ncRNA genes. This is crucial for advancing the development of ncRNA therapeutics and bringing them closer to clinical application (16, 85). Additional concerns, such as inadequate cellular or tissue penetration and specific delivery media, require consideration (16, 85, 86). Above all, future research in ncRNA-based therapeutics should focus on mitigating off-target effects while simultaneously enhancing immunogenicity, safety, stability, and customization (16).

In summary, ncRNA-based combination therapies may hold theoretical promise. However, several significant obstacles limit their clinical application. To tackle the issues of instability, off-target effects, and low transfection efficiency in ncRNA-based combination therapies, numerous carriers and systems for ncRNA have been suggested and extensively studied. These include different types of nanoparticles, ncRNA modification techniques, and the oncolytic adenovirus strategy (15). In the future, more strategies are expected to be implemented to address these clinical obstacles, and improved technologies should be utilized to investigate ncRNAs associated with osimertinib resistance with the aim of implementing them in clinical settings.
